# Prenatal polychlorinated biphenyl exposure is associated with decreased gestational length but not birth weight: archived samples from the Child Health and Development Studies pregnancy cohort

**DOI:** 10.1186/1476-069X-11-49

**Published:** 2012-07-20

**Authors:** Katrina L Kezios, Xinhua Liu, Piera M Cirillio, Olga I Kalantzi, Yunzhu Wang, Myrto X Petreas, June-Soo Park, Gary Bradwin, Barbara A Cohn, Pam Factor-Litvak

**Affiliations:** 1 Department of Epidemiology, Mailman School of Public Health, Columbia University, Room 1614, 722 West 168th St, New York, NY, 10032, USA; 2 Department of Biostatistics, Mailman School of Public Health, Columbia University, New York, NY, USA; 3 Child Health and Development Studies, Center for Research on Women’s and Children’s Health, Public Health Institute, Berkeley, CA, USA; 4 Department of Environment, University of the Agean, Mytilene, Greece; 5 Department of Toxic Substances Control, California Environmental Protection Agency, Berkeley, CA, USA; 6 Department of Laboratory Medicine, Harvard Medical School and Children's Hospital, Boston, MA, USA

**Keywords:** Endocrine disruptors, Polychlorinated biphenyls, Birth weight, Length of gestation, Prenatal exposures

## Abstract

**Background:**

Polychlorinated biphenyls (PCBs), known endocrine disruptors, were banned in 1979 but persist in the environment. Previous studies are inconsistent regarding prenatal exposure to PCBs and pregnancy outcomes. We investigated associations between prenatal exposure to PCBs and gestational length and birth weight.

**Methods:**

In a sample of 600 infants (born between 1960 and 1963) randomly selected from Child Health and Development Studies participants followed through adolescence we measured 11 PCB congeners in maternal post partum sera (within three days of delivery). Length of gestation was computed from the reported first day of the last menstrual period (LMP) and delivery date. Linear regression was used to estimate associations between PCB exposure and gestational age and birth weight, adjusting for potential confounders. PCBs were grouped according to hypothesized biological action (1b (sum of weak phenobarbital inducers), 2b (sum of limited dioxin activity), and 3 (sum of CYP1A and CYP2b inducers)) or degree of *ortho-* substitution (mono, di, tri). Secondary analyses examined associations between total PCB exposure and exposure to individual congeners.

**Results:**

Each unit increase in mono-*ortho* substituted PCBs was associated with a 0.30 week decrease (95% confidence interval (CI) -0.59, -0.016), corresponding to a 2.1 (95% CI −4.13, -0.11) day decrease in length of gestation. Similar associations were estimated for di-*ortho* substituted PCBs, (1.4 day decrease; (95% CI −2.9, 0.1)) and group 3 PCBs (0.84 day decrease; (95% CI −1.8, 0.11). We found similar associations in congener specific analyses and for the sum of congeners.

**Conclusions:**

Our study provides new evidence that PCB exposure shortens length of gestation in humans. This may have public health implications for population exposures.

## Background

Despite their ban in 1979, polychlorinated biphenyls (PCBs) remain ubiquitous environmental contaminants due to their chemical stability and lipophilic properties. PCBs are biphenyls with one to ten chlorine atoms; the degree of chlorination determines the stability and lipophilicity of any particular PCB congener. This structure also results in stereochemical resemblance to steroid hormones. In animal and human studies, PCBs cross the blood-placenta barrier [1,2]. Fetal exposure to PCBs may result in adverse pregnancy outcomes such as decreased length of gestation and decreased birth weight.

Both animal experiments and epidemiological studies suggest that the developing fetus is vulnerable to the effects of *in utero* PCB exposure. Early studies examining laboratory animals fed PCB rich diets report increased incidence of fetal resorption and abortion, reduced birth weight, and fetal growth retardation [3-5]. However, the dosing in these studies was much higher than the low-level environmentally relevant doses of today [6]. In human populations with high PCB exposure during pregnancy, such as poisoning incidents [7,8] or occupational exposure [9], reduced birth weight and gestational length are reported. Epidemiological evidence examining low-level environmental exposure is less consistent. Some studies find PCB exposure to be associated with decreased birth weight and length of gestation [10-13], although some but not all had small sample sizes – often fewer than 30 subjects [11,12]. In contrast, other studies find no evidence of association [14-18]. Further, in several early studies, a high percentage of values for a given PCB congener were under the limit of detection [11,17] making results less reliable and therefore difficult to interpret.

Varying methods used to characterize PCB exposure may account for some of the conflicting results. PCBs were generally used as mixtures (i.e. Arochlor). Many of the aforementioned studies characterize exposure either as the sum of all PCB congeners [14,15] or as individual congeners [14,18]. More recently, congener groupings were developed to summarize exposure based on either theoretical mode of action, reflecting the metabolic properties of the individual congeners, [19,20] or the degree of chlorine substitution as an indicator of the planarity of the molecule [21]. Additional grouping methods have been proposed, however it has been suggested that classification schemes based on either degree of chlorination or toxicological properties and enzyme induction may be the most useful [22]. Nevertheless exposure assessment is challenging because for many outcomes there is a lack of knowledge regarding exact biological mechanisms. We choose to focus on grouping according toxicological function and degree of chlorine substitution in primary analyses and explore total exposure and congener specific exposure in secondary analyses.

Here, we test the hypotheses that PCBs are associated with reduced length of gestation and reduced fetal growth. In secondary analyses, we also test whether associations, if any, are mediated by perturbed maternal thyroid function. We use data from the Child Health and Development Studies (CHDS) in which exposure was measured in stored maternal serum samples.

## Methods

### Study design

The CHDS is a prospective birth cohort designed to observe women, their pregnancies and their offspring [23]. The cohort recruited members of the Kaiser Health Plan who were receiving obstetric care at the Oakland California Kaiser Foundation Hospital and nearby East Bay clinics from 1959 through 1966. Women were enrolled at the confirmation of their pregnancies and followed through delivery. The CHDS includes data on 20,754 pregnancies and 19,044 live births. All live births were followed through at least age 5 years, with some subsets of children whose families remained in the San Francisco Bay area being observed through adolescence. At 12 months only 48 (0.2%) subjects were lost to follow up and at 5-years 89.4% were still under observation.

Detailed information on maternal and paternal characteristics was obtained through maternal interview. Maternal and pediatric medical records were abstracted to obtain information about maternal prenatal care measures, labor and delivery, and child’s serial growth (weight and height) through age 5. Serum samples were collected from the women during each trimester of pregnancy and postpartum. After fractionation, serum samples were frozen at −20°C, stored and archived. Subjects provided informed consent in accordance with practices in the 1960s.

The data in the present analysis are derived from a study of organochlorine compounds (OCs), thyroid hormone levels and neurocognitive outcomes at ages 5, 9–11 and 15–17. The study base for the present analysis is drawn from a subset of CHDS children who participated in neurocognitive examinations through successive follow-up at ages 5, 9–11 and 15–17 (n = 1752). Of these participants, we analyzed a sample of 600 infants (300 male, 300 female). The 600 were selected at random from the 500 males and 453 females who remained eligible after excluding subjects with inadequate second trimester sera (for the measurement of thyroid hormones), inadequate postpartum maternal serum (for the measurement of OCs) and subjects with missing gestational age. Pregnancy serum samples used for this analysis were drawn between 1960–1963.

### Laboratory analyses

All serum samples were stored at −20°C, with minimal thaw-refreeze cycles, until analyzed in 2007–2008. OCs were measured in sera collected immediately post partum (within three days of delivery) from the mother. Serum levels of 11 PCB congeners (66, 74, 99, 118, 138, 153, 170, 180, 187, 194 and 203) were measured. Serum levels of 1,1,1-trichloro-2,2-bis(*p*-chlorophenyl)ethane (*p,p′*-DDT), 1,1′-dichloro-2,2’-bis(*p*-chlorophenyl)ethylene (*p,p′-*DDE) and 1,1,1-trichloro-2-(*p*-chlorophenyl)-2-(*o*-chlorophenyl)-ethane (*o,p′-*DDT) were also measured. Assays were performed at the Environmental Chemistry Laboratory (ECL) of the California Department of Toxic Substances Control (n = 480) or at the Mount Sinai School of Medicine (MSSM. n = 120). Assay methods are detailed in a previous publication [24]. Serum triglyceride and cholesterol levels were measured in the laboratory of Dr. Nadir Rafai at Harvard Medical School.

Previous reports have established that a wide array of OCs are detectable in CHDS serum and measures between laboratories are comparable [25,26]. Inter batch coefficients of variations (CV) were calculated both for study subjects and quality control (QC) samples. A comparison of CV ratios (study subjects vs QC samples) between the two laboratories indicates that for all but two PCB congeners (66 and 99) both had a CV ratio above two [26], demonstrating that measurement error is small relative to natural variation found in the data. The limits of detection (LOD) for both laboratories ranged from 0.01 to 0.07 ng/mL and 94% of samples were above the respective LODs for PCB congeners. As in our previous work [27] PCB values below the LOD were used as measured rather than recoding them to the LOD.

Maternal thyroid function during pregnancy was assessed using FT4 and TSH measured in archived samples taken in the second trimester. All thyroid assays were performed at Children’s Hospital/Harvard Medical School. FT4 and TSH were measured using immunoassays on the Roche E Modular system (Roche Diagnostics, Indianapolis, IN). For FT4, the detection limit is 0.023 ng/dL and the day-to-day imprecision values at concentrations of 0.68, 1.64 and 3.94 ng/dL are 3.5, 3.3 and 6.6%, respectively. For TSH, the detection limit is 0.005 uIU/mL and the day-to-day imprecision values at concentrations of 0.084, 0.91 and 3.96 uIU/mL are 5.4, 2.1 and 1.8%, respectively. In a preliminary study (Factor-Litvak, unpublished data), we found that second trimester serum samples from pregnant women stored at −20 C for approximately 20 years had values of total T4 and TSH comparable to that of fresh sera.

### Classification of exposure variables

Individual PCB congeners were aggregated into group variables in two ways. First, measured PCBs were placed into three groups, according to their degree of chlorine substitution on the *ortho* position of the molecule PCB_mono_ (66, 74, 118), PCB_di_ (99, 138, 153, 170, 180) and PCB_tri_ (187, 203) [21]; we hypothesized that higher degrees of *ortho* chlorination (i.e. less coplanar molecules) would be more likely to act as thyroid hormone disruptors. Congeners were also grouped according to proposed biological mechanism [19]: Group 1b, those potentially estrogenic (187), Group 2b, those with limited dioxin-like potential (138, 170) and Group 3, those which inhibit CYP1A and CYP2B (99, 153, 180 and 203). In secondary analyses, exposure was defined as individual congeners and as the sum of all congeners. We also categorized all PCB measures into tertiles, based on the rank order, to explore the shape of the dose response relationships.

### Description of prenatal outcomes

Infant birth weight and length of gestation were extracted from medical records. Length of gestation was determined by mothers’ self-reported last menstrual period and recorded delivery date. There were 14 women in the sample with gestational length greater than 44 weeks; excluding these women did not change the findings.

Birth weight in grams and length of gestation in weeks were examined as continuous measures and were the primary outcomes in the study. We also calculated percentile birth weight for gestational age, using race stratified tables [28]. In a secondary analysis, we examined preterm delivery (delivery <37 weeks gestation) as an outcome.

### Description of potential covariates

Potential covariates included maternal race, smoking status, occupation, education, age, BMI, parity and infant sex. Where possible, covariates were analyzed as continuous variables in regression models (i.e. maternal age and BMI), although for descriptive tables all variables were expressed categorically. Categories of variables included maternal race (white, black, and mixed-race/other), smoking (never, former, and current), occupation (employed and house-wife/part-time worker), education (less than a high-school diploma, a high school diploma only, a high school diploma and some college or trade school, and 4-year college degree), age at study pregnancy (15–24, 25–29, 30–34, and ≥ 35 years), BMI (underweight (<18.5 kg/m^2^), normal weight (18.5-24.99 kg/m^2^), overweight (25–29.99 kg/m^2^), and obese (≥30 kg/m^2^)), parity (0, 1, 2, and ≥3) and infant sex (male and female). Pre-pregnancy measures of maternal weight were limited by sample size (N = 400) so BMI in our sample reflects weight of the mother at interview or first prenatal visit (N = 583). Pre-pregnancy and first visit BMIs were highly correlated since many women had early enrollment and/or early prenatal care (means for the two time periods were 22.2 vs 23.2 kg/m^2^, respectively; correlation coefficient: r = .94, p<0.0001). *p,p′-*DDE was also examined as a covariate, as it was in Longnecker, et al. [15].

### Statistical analysis

The distributions of all variables were examined. The variables with skewed distributions were transformed to reduce impact of extreme values. OC concentrations were transformed using the natural logarithmic function and TSH concentrations were transformed using the square root function. We performed all analysis both with non-transformed and natural log-transformed PCB exposures and the interpretation of results did not substantially differ; thus, we report in text the simpler results of analyses using non-transformed PCBs (however, see Additional file 1 Tables S3 and S4 for results of linear regression analyses using natural-log transformed PCB exposures). Correlations between individual PCB congeners, PCB groupings and *p,p′-*DDE were also examined using the Spearman’s rank correlation coefficients. Preliminary analyses compared the 600 infants selected for the study to those not selected using Chi- squares for categorical variables and t-tests for continuous variables.

We used multiple linear regression analysis to assess the associations between each exposure and outcome, with and without adjustment for potential confounders. Potential confounders were selected based on those previously reported to be associated with the outcome in the literature or those known to confound the relationship between PCBs and pregnancy outcomes. These included infant sex, maternal age, race, education, BMI, occupation, marital status, parity, smoking status and exposure to *p,p′-DDE*. All models adjusted for the lab (ECL vs. MSSM) in which PCBs were measured, and serum triglycerides and cholesterol (which were transformed using the natural logarithmic function) [29]. Potential covariates were included in a ‘core’ regression model if they were associated with the outcome. The core regression model did not include the exposure of interest. After exposure was included, covariates were deleted from final regression models if their exclusion resulted in an unsubstantial change (<0.5 standard error) in the estimated regression coefficient relating exposure to outcome. Final linear models for birth weight adjusted for the following covariates: maternal race, age, smoking status and BMI, infant sex, linear and quadratic terms for length of gestation centered at its mean. Linear models for gestational age adjusted birth weight percentiles were adjusted for maternal age, smoking status and BMI, and infant sex. Linear models for length of gestation were adjusted for maternal race, age, employment status and infant sex.

We also used Cox proportional hazards models to estimate the hazard ratio indicating the change in probability of giving birth on the next day per unit increase in exposure, with higher hazard ratio indicative of shorter gestational length.

We excluded subjects missing covariates (N = 21, 21, and 8 for the birth weight, birth weight percentile and length of gestation outcomes, respectively). For an additional 22 women, OC, total cholesterol and triglyceride concentrations were unable to be measured. These subjects did not differ from the remaining on important variables, including the outcomes. Further taking into consideration exclusion of the 14 participants with length of gestation greater than 44 weeks, final sample sizes for birth weight, birth weight percentile and length of gestation models were 556, 543, and 543 respectively.

We examined whether the associations between PCBs and birth outcomes were mediated by maternal thyroid function using linear regression analysis [30]; that is we examined whether maternal thyroid function was in the pathway between PCB exposure and birth outcomes. In these analyses, FT4 and TSH were included separately in the regression models. We considered the following as evidence for mediation: (1) associations between FT4 and/or TSH and PCBs, (2) associations between FT4 and/or TSH and length of gestation and/or birth weight, and (3) reductions in the estimated regression coefficient for PCBs when FT4 and/or TSH is added to the model.

Final analyses examined sex specific associations between exposures and each birth outcome as reported previously [15,31].

All analyses were performed using SAS version 9.2.

## Results

Our sample had a mean birth weight of 3387 g and mean gestational age of 40.2 weeks (Table 1). Comparisons between the 600 randomly selected subjects and those not selected show no significant differences in most of the relevant variables (birth weight, number of previous live births, percent male infants, mothers’ education at birth, mothers’ smoking status during pregnancy). However, gestation in those selected for analysis was significantly longer (by 3 days) compared to those not selected (p<0.001). In Table 2, the distributions of maternal concentrations of PCB groupings and individual PCB are displayed. Nearly all (94%) congeners were well above the limits of detection and the range of concentrations of PCB groupings and individual congeners were comparable to other studies performed at the time [32]. Concentrations of PCBs were higher in older women and those with a college education and lower in white mothers (Table 3). PCB concentrations did not decline with parity, reflecting continuing exposure during the 1960s; we also note that few women (38.7%) in this cohort breast fed, which is an efficient way to reduce the OC burden in new mothers. No significant associations were found between any PCB exposure variable and birth weight (Table 4) or birth weight percentile (data not shown) either before or after adjustment for potential confounders. Further, we did not find significant associations between PCB exposure and preterm delivery (Additional file 1 Table S1), though the number of preterm deliveries in our sample was small (N = 21; mean gestation for preterm deliveries: 35.8 ± 1.05 weeks; range: 32.6 – 36.9 weeks).

**Table 1:** Comparison of key variables at study baseline between those selected into the study and those not selected from the Adolescent Study

**Variable**	**Selected (N = 600)**			**Not Selected (N = 1420)**				
	**N**	**Mean**	**Std**	**N**	**Mean**	**Std**	**p-value**	
Birth weight (g)	600	3387.8	493.3	1420	3353.7	533.0	0.92	
Gestation (days; LMP to term)	600	282.1	12.7	1404	279.1	16.3	<.001	
Number of Previous Live Births	600	1.7	1.7	1420	1.9	1.8	0.09	
	**N**	**%**		**N**	**%**		**p-value**	
Male	300	50.0		703	49.5		0.84	
Mother Education at Child’s Birth								
< High School	74	12.3		169	11.9		0.58	
High School Graduate	202	33.7		477	33.6			
Some College/Trade School	200	33.3		444	31.3			
College Graduate or Higher	123	20.5		329	23.2			
Missing	1	0.2		1	0.1			
Maternal Smoking During Pregnancy								
Never Smoked	315	52.5		662	46.6		0.16	
Smokes Now	180	30.0		490	34.5			
Smoked Until Current Pregnancy	40	6.7		119	8.4			
Former Smoker	61	10.2		136	9.6			
Missing	4	0.7		13	0.9			

Data are presented as number, (*N*), mean and standard deviation (*std*).

**Table 2:** Distribution of maternal PCB concentrations

**Exposure category**^+^	**No.**	**Percentile distributions**			**Mean ± SD**	**Min**	**Max**
		**25**^th^	**50**^th^	**75**^th^			
∑PCB_total_^1^	578	2.37	2.96	3.82	3.30 ± 1.53	0.57	12.29
∑PCB_mono_^2^	578	0.52	0.68	0.92	0.81 ± 0.52	0.13	6.82
∑PCB_di_^3^	578	1.57	2.00	2.60	2.23 ± 1.05	0.40	8.98
∑PCB_tri_^4^	578	0.18	0.24	0.32	0.26 ± 0.15	0.02	1.90
∑PCB_1b_^5^	578	0.11	0.15	0.20	0.17 ± 0.09	0.02	0.83
∑PCB_2b_^6^	578	0.56	0.72	0.96	0.82 ± 0.44	0.17	3.85
∑PCB_3_^7^	578	1.08	1.37	1.77	1.51 ± 0.66	0.23	5.29
PCB66	578	0.05	0.08	0.13	0.15 ± 0.23	0.01	2.05
PCB 74	578	0.12	0.17	0.24	0.20 ± 0.17	0.01	3.07
PCB 99	578	0.12	0.18	0.25	0.21 ± 0.15	0.01	1.18
PCB 118	579	0.31	0.40	0.53	0.46 ± 0.26	0.08	2.47
PCB 138	579	0.45	0.57	0.77	0.66 ± 0.36	0.13	3.23
PCB 153	579	0.50	0.65	0.85	0.73 ± 0.35	0.14	2.95
PCB 170	578	0.10	0.14	0.19	0.16 ± 0.09	0.01	0.72
PCB 180	579	0.33	0.44	0.56	0.47 ± 0.2	0.08	1.71
PCB 187	578	0.11	0.15	0.20	0.09 ± 0.09	0.02	0.83
PCB 194	459	0.06	0.08	0.11	0.09 ± 0.05	0.06	0.48
PCB 203	578	0.06	0.09	0.13	0.10 ± 0.08	0.01	1.27
FT4 (ng/dL)	600	1.13	1.23	1.38	1.26 ± 0.21	0.76	2.46
TSH (μIU/mL)	600	0.86	1.29	1.92	1.53 ± 1.16	0.005	12.55

^+^All PCBs are in units of ng/mL.

^1^PCB congeners summed into PCB_total_: all congeners with LOQ >90% except PCB_194_ due to large number of missing values.

^2^PCB congeners summed into PCB_mono_: 66, 74, 118.

^3^PCB congeners summed into PCB_di_: 99, 138, 153, 170, 180.

^4^PCB congeners summed into PCB_tri_: 187, 203.

^5^PCB congeners summed into PCB_1b_: 187.

^6^PCB congeners summed into PCB_2b_: 138, 170.

^7^PCB congeners summed into PCB_3_: 99, 153, 180, 203.

^#^LODs were different for the two labs; the most conservative LODs are reported in the table (from the MSSM lab, n = 120). LODs for the ECL (n = 460) range from 0.01 to 0.03, with 99% of samples above LODS for the various congeners. LODs are reported in ng/mL.

**Table 3:** **Concentrations of PCB groupings (mean ± SD) by study sample characteristics (n = 578)**^**1**^

**Sample Characteristics**	**PCB**_**total**_	**PCB**_**mono**_	**PCB**_**di**_	**PCB**_**tri**_	**PCB**_**1b**_	**PCB**_**2b**_	**PCB**_**3**_
Total sample	3.30 ± 1.53	0.81 ± 0.52	2.23 ± 1.05	0.27 ± 0.15	0.17 ± 0.09	0.82 ± 0.44	1.51 ± 0.66
Maternal race							
White^§^	3.21 ± 1.34	0.80 ± 0.46	2.15 ± 0.90	0.26 ± 0.14	0.16 ± 0.08	0.78 ± 0.37	1.47 ± 0.58
Black	3.32 ± 1.79	0.74 ± 0.41	2.31 ± 1.35	0.30 ± 0.17	0.18 ± 0.11	0.89 ± 0.57	1.51 ± 0.82
Other	4.02 ± 2.07^*^	1.06 ± 0.92^*^	2.64 ± 1.30^*^	0.27 ± 0.16	0.21 ± 0.11^*^	0.99 ± 0.53^*^	1.76 ± 0.81
Sex of infant							
Male^§^	3.26 ± 1.58	0.79 ± 0.58	2.21 ± 1.03	0.26 ± 0.17	0.16 ± 0.09	0.82 ± 0.42	1.49 ± 0.65
Female	3.35 ± 1.49	0.83 ± 0.45^*^	2.24 ± 1.07	0.27 ± 0.13	0.17 ± 0.09	0.82 ± 0.45	1.52 ± 0.66
Maternal smoking status							
Never^§^	3.33 ± 1.57	0.82 ± 0.45	2.25 ± 1.13	0.27 ± 0.13	0.17 ± 0.09	0.84 ± 0.48	1.51 ± 0.70
Current	3.28 ± 1.29	0.79 ± 0.48	2.22 ± 0.82	0.27 ± 0.13	0.16 ± 0.08	0.80 ± 0.33	1.53 ± 0.55
Former	3.26 ± 1.85	0.83 ± 0.75	2.17 ± 1.17	0.27 ± 0.22	0.16 ± 0.10	0.80 ± 0.49	1.47 ± 0.74
Maternal occupation							
Employed	3.25 ± 1.43	0.79 ± 0.46	2.20 ± 0.96	0.26 ± 0.13	0.16 ± 0.09	0.81 ± 0.39	1.49 ± 0.62
Housewife/Part-time^§^	3.32 ± 1.59	0.82 ± 0.55	2.23 ± 1.10	0.27 ± 0.16	0.17 ± 0.09	0.83 ± 0.47	1.51 ± 0.68
Maternal education^†^							
No HS diploma	3.16 ± 1.47	0.76 ± 0.40	2.14 ± 1.03	0.26 ± 0.16	0.17 ± 0.10	0.80 ± 0.42	1.43 ± 0.67
HS diploma only^§^	3.10 ± 1.26	0.76 ± 0.37	2.10 ± 0.91	0.25 ± 0.13	0.15 ± 0.09	0.77 ± 0.37	1.42 ± 0.57
HS + some college	3.34 ± 1.62	0.79 ± 0.45	2.28 ± 1.17	0.28 ± 0.17	0.17 ± 0.09	0.85 ± 0.50	1.54 ± 0.72
College graduate	3.64 ± 1.77	0.96 ± 0.80^*^	2.39 ± 1.05^*^	0.29 ± 0.13	0.17 ± 0.08	0.88 ± 0.44	1.63 ± 0.66^*^
Maternal age categories							
15-24	3.03 ± 1.32	0.74 ± 0.38	2.07 ± 0.97	0.23 ± 0.12	0.14 ± 0.09	0.76 ± 0.42	1.39 ± 0.59
25-29^§^	3.34 ± 1.56	0.79 ± 0.44	2.28 ± 1.09	0.27 ± 0.14	0.17 ± 0.09	0.84 ± 0.45	1.54 ± 0.70
30-34	3.42 ± 1.65	0.84 ± 0.50	2.31 ± 1.18	0.27 ± 0.12	0.17 ± 0.08	0.86 ± 0.51	1.55 ± 0.72
≥35	3.48 ± 1.59	0.90 ± 0.75	2.26 ± 0.91^*^	0.32 ± 0.20	0.19 ± 0.10	0.82 ± 0.35	1.56 ± 0.60
Maternal BMI categories							
Underweight	3.17 ± 1.24	0.68 ± 0.32	2.21 ± 0.88	0.28 ± 0.15	0.17 ± 0.09	0.78 ± 0.34	1.54 ± 0.56
Normal^§^	3.42 ± 1.62	0.84 ± 0.57	2.30 ± 1.10	0.28 ± 0.16	0.17 ± 0.09	0.85 ± 0.46	1.56 ± 0.69
Over-weight	3.02 ± 1.35	0.73 ± 0.36	2.05 ± 0.99	0.24 ± 0.12	0.15 ± 0.08	0.76 ± 0.41	1.38 ± 0.62
Obese	3.01 ± 1.42	0.85 ± 0.45	1.93 ± 0.91	0.23 ± 0.14	0.15 ± 0.09	0.72 ± 0.39	1.29 ± 0.59^*^
Parity							
0^§^	3.21 ± 1.49	0.76 ± 0.41	2.20 ± 1.06	0.25 ± 0.14	0.16 ± 0.09	0.81 ± 0.45	1.48 ± 0.65
1	3.22 ± 1.58	0.83 ± 0.69	2.13 ± 0.96	0.25 ± 0.11	0.16 ± 0.07	0.77 ± 0.39	1.45 ± 0.61
2	3.46 ± 1.33	0.83 ± 0.43	2.34 ± 0.89^*^	0.30 ± 0.13^*^	0.18 ± 0.09	0.86 ± 0.36	1.59 ± 0.58
≥3	3.36 ± 1.66	0.83 ± 0.47	2.26 ± 1.21	0.28 ± 0.19	0.17 ± 0.10	0.85 ± 0.51	1.52 ± 0.76

^1^Differences in PCB levels within categories of maternal covariates were evaluated using analysis of variance with Bonferroni corrections.

^†^HS = high-school.

^§^reference category.

*p<0.05.

**Table 4:** Associations between non-transformed PCB exposure and birth weight

	**Unadjusted**		**Adjusted**		**Further adjusted for*p,p′*-DDE**	
	**b**_unadjusted_^1^	**95%CI**	**b**_adjusted_^2^	**95% CI**	**b**_3adjusted_	**95%CI**
**PCB groupings**						
PCB_total_	−4	(−33, 25)	11	(−15, 38)	16	(−15, 46)
PCB_mono_	24	(−63, 110)	36	(−43, 115)	41	(−42, 125)
PCB_di_	−16	(−57, 26)	11	(−27, 49)	17	(−27, 60)
PCB_tri_	77	(−219, 374)	162	(−115, 439)	215	(−96, 527)
PCB_1b_	−26	(−508, 456)	243	(−210, 696)	341	(−180, 863)
PCB_2b_	−26	(−124, 72)	34	(−56, 124)	47	(−54, 148)
PCB_3_	−24	(−91, 42)	17	(−44, 79)	26	(−45, 98)
**Individual PCBs**						
PCB 66	22	(−204, 247)	27	(−176, 231)	31	(−177, 239)
PCB 74	57	(−187, 302)	134	(−89, 356)	146	(−84, 376)
PCB 99	−137	(−431, 158)	9	(−256, 275)	15	(−263, 294)
PCB 118	51	(−117, 218)	55	(−98, 209)	62	(−99, 222)
PCB 138	−16	(−136, 104)	52	(−58, 162)	68	(−55, 191)
PCB 153	−61	(−187, 65)	20	(−95, 136)	29	(−102, 160)
PCB 170	−245	(−719, 228)	56	(−379, 491)	86	(−401, 574)
PCB 180	0.24	(−217, 218)	112	(−89, 313)	163	(−75, 400)
PCB 187	−26	(−509, 456)	243	(−210, 696)	341	(−180, 863)
PCB 194	23	(−1005, 1051)	311	(−669, 1292)	601	(−468, 1669)
PCB 203	339	(−245, 924)	271	(−271, 812)	310	(−259, 878)

^1^Parameter estimate for non-transformed PCB measure (adjusted: laboratory, natural log-transformed serum triglycerides and cholesterol).

^2^Parameter estimate for non-transformed PCB measure (adjusted: maternal race, age, smoking status and BMI, infant sex, length of gestation (centered), quadratic length of gestation (centered), laboratory and natural log-transformed serum triglycerides and cholesterol).

^3^Parameter estimate for non-transformed PCB measure (adjusted: *p,p′-*DDE, maternal race, age, smoking status and BMI, infant sex, length of gestation (centered), quadratic length of gestation (centered), laboratory and natural log-transformed serum triglycerides and cholesterol).

We found consistent reductions in length of gestation related to PCB exposure, regardless of exposure categorization (Table 5). For example, decreases in length of gestation were found for mono-*ortho* substituted PCBs (0.30 week decrease (95% CI −0.59, -0.016)) for each unit change in the exposure variable. Decreases of slightly smaller magnitude were observed for di-*ortho* substituted PCBs (0.12 week decrease (95% CI −0.25, 0.017)), and PCBs in group 3 (0.20 week decrease (95% CI −0.42, 0.014)). For the sum of PCBs, each unit change of *in utero* exposure was associated with an adjusted reduction of approximately 0.10 weeks of gestation (95% CI −0.19, -0.0016). Consistent results were found in the analysis using Cox- proportional hazards models (Additional file 1 Table S2a). For example, the covariate-adjusted hazard ratio for delivery on the next day for each unit change of di-*ortho* substituted PCB concentration was 1.07 (95% CI 1.00, 1.16). Lastly, results were essentially unchanged when natural log-transformed PCB variables are used as continuous exposure in regression models (Additional file 1 Tables S3 and S4).

**Table 5:** Associations between non-transformed PCB exposure and length of gestation

	**Unadjusted**		**Adjusted**		**Further adjusted for*p,p′*-DDE**	
	**b**_unadjusted_^1^	**95%CI**	**b**_adjusted_^2^	**95% CI**	**b**_3adjusted_	**95%CI**
**PCB groupings**						
PCB_total_	−0.096^*^	(−0.19, -0.0021)	−0.096^*^	(−0.19, -0.0016)	−0.087	(−0.20, 0.022)
PCB_mono_	−0.26^+^	(−0.54, 0.025)	−0.30^*^	(−0.59, -0.016)	−0.27^+^	(−0.57, 0.031)
PCB_di_	−0.13^+^	(−0.27, 0.0035)	−0.12^+^	(−0.25, 0.017)	−0.099	(−0.25, 0.057)
PCB_tri_	−0.41	(−1.38, 0.55)	−0.54	(−1.53, 0.44)	−0.31	(−1.43, 0.80)
PCB_1b_	−1.17	(−2.76, 0.42)	−1.09	(−2.72, 0.54)	−0.75	(−2.63, 1.14)
PCB_2b_	−0.29^+^	(−0.61, 0.031)	−0.24	(−0.56, 0.081	−0.18	(−0.55, 0.18)
PCB_3_	−0.21^+^	(−0.42, 0.011)	−0.20^+^	(−0.42, 0.014)	−0.18	(−0.43, 0.077)
**Individual PCBs**						
PCB 66	−0.70^+^	(−1.42, 0.0095)	−0.89^*^	(−1.60, -0.18)	−0.83^*^	(−1.56, -0.11)
PCB 74	−0.56	(−1.35, 0.24)	−0.58	(−1.38, 0.22)	−0.47	(−1.30, 0.36)
PCB 99	−0.59	(−1.55, 0.37)	−0.48	(−1.43, 0.48)	−0.32	(−1.32, 0.68)
PCB 118	−0.29	(−0.84, 0.26)	−0.33	(−0.88, 0.23)	−0.25	(−0.82, 0.33)
PCB 138	−0.32	(−0.71, 0.071)	−0.26	(−0.65, 0.13)	−0.18	(−0.62, 0.26)
PCB 153	−0.42^*^	(−0.83, -0.0093)	−0.38^+^	(−0.79, 0.035)	−0.32	(−0.79, 0.15)
PCB 170	−1.58^*^	(−3.12, -0.043)	−1.54^*^	(−3.07, -0.0027)	−1.37	(−3.10, 0.36)
PCB 180	−0.57	(−1.28, 0.13)	−0.74^*^	(−1.46, -0.017)	−0.69	(−1.55, 0.18)
PCB 187	−1.17	(−2.76, 0.42)	−1.09	(−2.72, 0.54)	−0.75	(−2.63, 1.14)
PCB 194	−1.18	(−4.55, 2.19)	−2.32	(−5.69, 1.06)	−2.15	(−5.87, 1.57)
PCB 203	0.065	(−1.83, 1.96)	−0.55	(−2.47, 1.37)	−0.16	(−2.19, 1.86)

^1^Parameter estimate for non-transformed PCB measure (adjusted: laboratory, natural log-transformed serum triglycerides and cholesterol).

^2^Parameter estimate for non-transformed PCB measure (adjusted: maternal race, age, employment status and infant sex, laboratory and natural log-transformed serum triglycerides and cholesterol).

^3^Parameter estimate for non-transformed PCB measure (adjusted: *p,p′-*DDE, maternal race, age, employment status and infant sex, laboratory and natural log-transformed serum triglycerides and cholesterol).

^+^significant at 0.05<p<0.10; ^*^significant at p<0.05.

**Figure 1:**
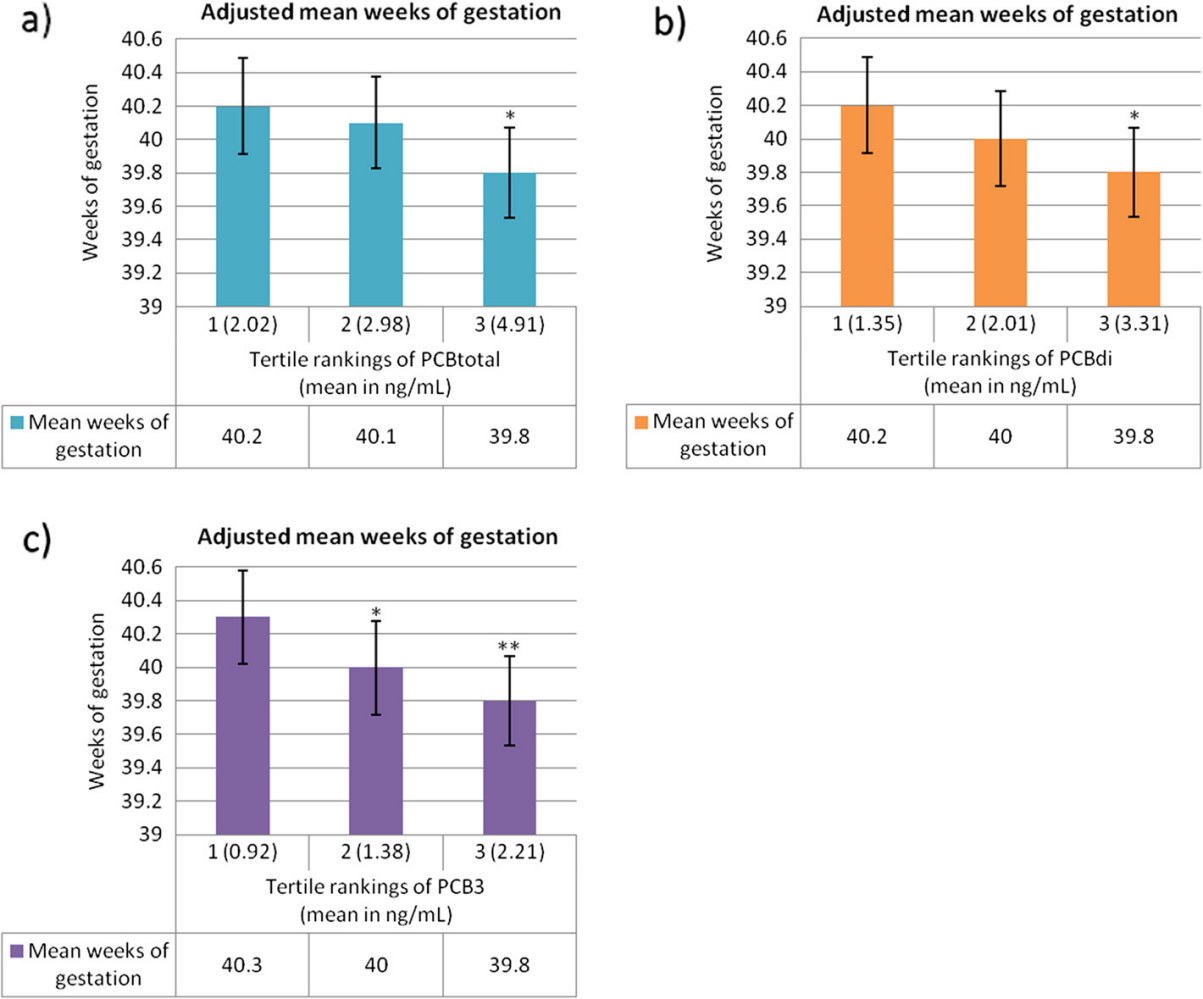
**Adjusted mean length of gestation in tertiles of PCB group exposure.** Adjusted mean weeks of gestation and 95% confidence intervals for tertiles of PCB groupings achieving statistical significance at p<0.05 when comparing the middle or highest tertile to the lowest (reference): PCB_total_ (1**a**.), PCB_di_ (1**b**.) and PCB_3_ (1**c**.). Linear regression models were adjusted for maternal race, age, employment status and infant sex, laboratory and log-transformed serum triglyceride and cholesterol. For PCB group 3, adjusted mean length of gestation decreases significantly from the lowest category of exposure to both the middle (p<0.05) and to the highest category of exposure (p<0.01). Tertile cut-points for total PCBs were 0.57-2.51, 2.52-3.50, and 3.51-12.29 ng/mL for tertiles 1, 2 and 3, respectively. Tertile cut-points for the di-*ortho* substituted PCB group were 0.40-1.69, 1.69-2.34, and 2.34-8.98 for tertiles 1, 2 and 3, respectively. Tertile cut-points for PCB group 3 were 0.23-1.17, 1.18-1.61, and 1.61-5.29 for tertiles 1, 2 and 3, respectively. ^*^p<0.05. ^**^p<0.01.

After collapsing exposure into tertiles, we found that the magnitude of decreased length of gestation was most pronounced comparing the highest tertile of PCB exposure to the lowest tertile of exposure in linear regression models (Figure 1). Results from the Cox proportional hazards models were consistent (Additional file 1 Table S2b). For example, the covariate-adjusted hazard ratio for delivery on the next day comparing the highest tertile of di-*ortho* substituted PCB concentration to the lowest was 1.29 (95% CI 1.04, 1.59); comparing the second highest tertile of di-*ortho* substituted PCB concentration to the lowest gives a hazard ratio of 0.99 (95%CI 0.80, 1.23).

We also performed separate analyses relating birth outcomes with exposure to OC pesticides and their metabolites (*p,p′*-DDT*, o,p′*-DDT and *p,p′*-DDE), and found an association only for *p,p′-*DDE and gestational length (unpublished observations). Thus, secondary analyses were run adding *p,p′-*DDE to the models as a potential confounder. Regression results were not substantially attenuated and remained consistently in the negative direction, albeit of lesser statistical significance (Table 5).

We found no evidence of mediation by maternal FT4 or TSH levels; neither FT4 nor TSH were associated with exposure and neither altered the parameter estimate when included in the regression model as a mediator. Likewise, there was no difference in results of gender specific analyses. Tests for interaction of exposure by infant sex on the outcomes using regression analysis were negative (data not shown).

## Discussion

Overall, we found evidence of an inverse association between maternal exposure to PCBs and length of gestation. The association was most prominent for the highest levels of PCB exposure. The direction of associations for PCB groupings, individual congeners and the total sum of all congeners was highly consistent which increases our confidence in the results. While various grouping methods have been proposed, the consistency in parameter estimates in our study – regardless of exposure metric used – suggests that PCBs in our sample operate to decrease gestation via a similar underlying mechanism regardless of hypothesized differences in mechanism of action. We observed no associations between birth weight and prenatal exposure to PCBs. Previous studies relating PCBs and birth outcomes are inconsistent. Patandin et al. [10] and Fein et al. [11] find inverse associations between PCB exposure and birth weight; Hertz-Picciotto et al. [31] find gender specific inverse associations, with only boys showing decreased birth weight and only girls showing shorter gestational age. Relationships between preterm delivery (defined as <37 weeks) and OC exposure also have been reported [11,12,33]. However, there are equally as many studies reporting no such associations [14-18]. Several factors may account for these inconsistencies. First, associations may only be evident where exposure is high; for example, associations with birth weight are more often reported in study populations with higher background exposure to PCBs [34]. Second, different PCB congeners are measured and in some cases, the congeners are classified differently. Differences in the toxicities of particular congener mixtures may contribute to inconsistent results and make comparisons between studies difficult [15], particularly in conjunction with different background levels of exposure. Finally, intrinsic differences in susceptibility to PCB toxicity in particular populations may result in discrepant findings.

Our results for length of gestation are in agreement with some previous studies, but not with others. One early study reported a significant decrease in gestational age comparing cord serum PCB concentrations above the limit of detection (≥3 ng/mL, n = 75) to those below (<3 ng/mL, n = 166). However, only congeners present in Arochlor 1260 were measured, and the majority of subjects had levels below the limit of detection; mean cord serum PCB levels were 2.5 ± 1.9 ng/mL [11]. In a New York based case–control study of 20 preterm deliveries and matched controls (delivered between October 1990 and August 1993), no association was observed between exposure to 12 PCB congeners (82/151, 118, 153, 141, 138, 187, 183, 174, 156, 180, 170, 203) and preterm delivery [14]. This study too was hindered by detection limits (the authors state that many PCB measurements were below the limit of detection). Median levels of congener 180 were 0.17 ng/mL (range: 0.05, 0.45) for cases and 0.19 (range: 0.08, 0.62) for controls, whereas our study reported a median PCB 180 value of 0.44 (range: 0.08, 1.71).

More recently, Wolff et al. [16] assessed PCB exposure in maternal plasma in the Children’s Environmental Health Study, an ethnically diverse New York cohort established between 1998 and 2002. Exposure was examined as the sum of congeners 118, 138, 153 and 180, similar to our di-*ortho* substituted PCB group (except for PCB 170). They found no associations between PCBs and either birth weight or length of gestation in a sample of 404 participants; however, exposure levels were very low (median total PCB < 1 ng/mL). The median value of the di-*ortho* substituted PCB group in our sample was 2.00 ng/mL, and over 95% of our sample had a concentration above 1 ng/mL (range: 0.40, 8.98 ng/mL); exclusion of congener 170 (median: 0.14 ng/mL; range: 0.01, 0.72 ng/mL) from our group does not alter this comparison. Mean total PCB concentration in our sample (2.96 ng/mL) is similar to that found by Longnecker et al. [15] who examined the association between PCB exposure (median total PCBs = 2.8 ng/ml) and birth outcomes in the National Collaborative Perinatal Project (NCPP), a prospective cohort of pregnant women recruited from 1959–1965. These investigators found unadjusted associations between maternal third trimester total PCB (highest quartile, ≥ 4 ng/ml, compared to the lowest quartile, < 2 ng.ml) and preterm birth (n = 132 vs n = 902 normal deliveries). After adjustment for *p,p′*-DDE and other potential confounders, the odds ratio diminished to 1.1 (95% CI 0.6, 2.2). We note that few pregnancies in our sample were preterm, making it difficult to confirm this result.

Our results for birth weight differ from other large samples in cohorts established around the same time. In the Longnecker et al. [15] study described above, associations between prenatal PCB exposure (sum of the congeners measured) and small-for-gestational age (SGA) infants were found and these associations were more pronounced among boys. In our study we saw no associations with birth weight or birth weight percentiles and no associations by sex.

In a different sample of infants drawn from the CHDS, Hertz-Picciotto et al. [31] found an overall suggestion of reduced birth weight with higher PCB exposure (comparing the 90^th^ percentile to the 10^th^ percentile), which was more evident among boys. Exposure was examined as the sum of nine individual PCB congeners, with the majority overlapping with the ones we measured (118, 138, 153, 170, 180 and 187). We repeated our adjusted birth weight analysis using this method and found a non-significant *increase* in birth weight comparing the 90^th^ percentile of exposure to the 10^th^: β = 138 g (95% CI −76, 351) and no gender differences. We also note that while Hertz-Picciotto et al. also selected mother-infant pairs from the CHDS, their sampling strategy was much different from ours. To test their primary hypotheses, they selected infants with adverse neurocognitive and neurosensory deficits. Because these outcomes may be related to birth weight, their results may not be surprising. Further, several key variables differed in their mother-infant pairs compared to the CHDS source population, including maternal race (study population more likely to be African-American), maternal occupation (less likely to be a professional and more likely to be a secretary/clerical worker), place of birth (more likely to be born in the Southeastern US), maternal alcohol consumption (more likely to consume low levels), maternal hypertension and pre-eclampsia (more likely to be diagnosed), medications potentially related to intra-uterine growth (less likely to be prescribed) and father’s occupation and education (less likely to be a professional or college graduate).

A recent meta-analysis of 15 studies in 12 European cohorts examined the association between PCB 153 and pregnancy outcomes including birth weight adjusted for gestational age [35]. All cohorts were established between 1990 and 2008 and thus had lower exposure levels than in the CHDS; exposure was measured in maternal and cord blood samples and in breast milk. PCB 153 was used as the primary measure of exposure. Low-level exposure to PCB 153 was associated with a 150 g decrease (95% CI −250, -50) in birth weight for each 1-ng/mL increase in PCB 153 [35]. Although not statistically significant, we found PCB 153 to be associated with decreased birth weight in unadjusted analyses (β = −61 g 95% CI −187, 65), however once adjusted for covariates the negative association disappeared (Table 4). Although the percentage of infants with birth weight <2500 g was not provided in the manuscript, birth weight ranged from 610 to 5500 g across the 7762 subjects included in analyses (median birth weight across the 15 studies ranged from 3210 to 3750 g). Most infants (98%) in our sample had birth weights above 2500 g and our median birth weight was 3374 g.

We originally hypothesized that PCB congeners associated with thyroid disruption (i.e. di- and tri-*ortho* substituted) would demonstrate stronger associations with measures of fetal growth and gestational age. However, our results demonstrate similar associations for all groupings of PCBs as well as single congeners. Thus, these results, along with the lack of evidence supporting mediation, do not support our hypothesis that reductions in fetal growth are due to the thyroid disrupting effects of PCBs.

Our study has many strengths. First, data were collected prospectively, avoiding recall bias and problems with temporality. High participation rates among those invited to join the full CHDS study reduced the potential for selection biases. We randomly selected our sample from those with successive follow-up through adolescence and with available serum, and those selected did not appreciably differ for many maternal characteristics from those who were not. Length of gestation did differ between selected and non-selected participants and was three days longer in the selected sample, likely making it more difficult to find associations with reduced gestation. To nullify findings observed in the selected group, shorter gestation infants in the non-selected sample would need to have much lower PCB concentrations.

Second, the expected relationships between known risk factors and adverse pregnancy outcomes (ex. smoking and decreased birth weight) are present in our sample, thus supporting the predictive validity of our data. In addition, we controlled for maternal characteristics known to be associated with PCB exposure or gestation in our sample. Inclusion of other known risk factors (maternal BMI, smoking, parity) did not significantly alter results (data not shown). Thus, confounding by other maternal characteristics is unlikely to be responsible for our findings.

Third, the wide range of exposure allowed examination of the association across PCB exposures from low to relatively high. Indeed, we found the significant difference in the birth outcomes between the exposure groups of the highest tertile and the lowest tertile. We were also able to discern whether the association varied by type of PCB.

Fourth, our outcomes were considered as continuous variables, rather than dichotomizing birth weight at 2500 g or length of gestation at 37 weeks, which we believe has the statistical advantage of avoiding the implied assumption of heterogeneity or risk within dichotomies of birth outcomes [36].

Fifth, we found consistency in results using both linear regression analysis and distribution free Cox proportional hazards analysis. We believe the Cox proportional hazards analysis is more useful than, in our case, a low-powered analysis of preterm deliveries.

A particular strength of this study is that we were able to test whether thyroid disruption mediates PCB effects on length of gestation. The proposed mechanism is likely due to the competitive binding by certain OCs to transthyretin (TTR), the transport protein that carries T4 from maternal circulation to the placenta [37]. There is some evidence that PCBs can perturb thyroid function [30] though there are also studies that do not support this hypothesis [38]. However, we did not find any evidence of mediation by maternal thyroid function in either the birth weight or length of gestation analyses, suggesting that PCBs impact fetal development via a different mechanism.

One possible explanation for our results, especially those relating to PCB group 3, concerns the possible relationship between these PCBs and cytochrome P450s, which may activate toxic or carcinogenic compounds [39]. A review article from 2011 indicates that CYP1A1 (a cytochrome P450 enzyme) expression is up-regulated through the ligand-activated aryl hydrocarbon receptor (AhR) [40]. Increased CYP1A1 activity through activated AhR in placentas of smokers is associated with pregnancy complications, including premature birth and risk of low birth weight [40].

Our study also has several limitations. First, we were missing either laboratory data or data on covariates on approximately 10% of our sample. However, comparisons of subjects with missing data to those without missing data did not show appreciable differences. Serum samples from these women were collected between 1960 and 1963 [41] before PCBs were banned, which provides the opportunity to examine a wide range of exposure. However, it is unknown whether lower levels of exposure currently observed in contemporary samples [42] have the same effects. Thus, we may not be able to extrapolate these findings to the current exposure levels. On the other hand, our population allows us to explore relationships that would be impossible to assess in contemporary serum. Regardless, this study supports proof of concept that PCBs can impact gestation.

Additionally, postpartum sera were used for the measurement of OCs. This was for a practical reason, as more sera were available for this time point. However, because OCs have a long half-life in adipose tissue we do not expect levels to vary during pregnancy. Indeed, Longnecker et al. found that OC levels measured in maternal blood collected serially in each trimester of pregnancy and early postpartum were highly correlated with minimal differences [43].

There may be concerns about multiple testing, however our analyses were specified *a priori* and analyses that were secondary or exploratory were stated as such. Although control for *p,p*′-DDE did not meaningfully alter our findings, we cannot rule out the possibility of confounding by a co-variable or other environmental contaminant that we did not measure. For instance, since the sera samples were stored at -20° C, we could not measure n-3 or n-6 fatty acids, also found in sea food, and which are beneficial to pregnancy outcomes.

Finally, although we do not have evidence to support that higher chlorinated PCBs have greater associations with fetal growth, we note that, in part, this can be attributed to the high correlations between the individual PCB congeners (Additional file 1 Tables S5a-c). Spearman correlation coefficients ranged from 0.57 (mono-*ortho* substituted and group 1b) to 0.98 (di-*ortho* substituted and group 3) between PCB groupings (Additional file 1 Table S5a.) and from 0.26 to 0.91 between individual congeners (Additional file 1 Table S5b.). Further, we considered the internal consistency among the PCB congeners by calculating Cronbach’s alpha. High internal consistency was found within the group of di-*ortho* substituted PCBs as well as group 2b PCBs (Cronbach’s alpha: 0.94 and 0.91, respectively) and moderate internal consistency was found for mono- and tri-*ortho* substituted PCBs and for group 3 PCBs (Cronbach’s alphas of 0.69, 0.76 and 0.81, respectively). This suggests that exposure to all congeners was relatively universal in the CHDS.

## Conclusions

Our results have public health implications for infant health, and potentially later adult health. Although seemingly small for an individual, in our sample a population shift in gestational age by age by a 1 and ½ day decrease would theoretically change the population mean from 280 days to 278.5 days, and result in a shift in the proportion of preterm births from 3.5% to 4.7%. Further, we note that gestational lengths in the 37 to 39 week range are also associated with increased morbidity in other studies [44]. Our results also may further inform current research on more contemporary endocrine disrupting compounds, such as brominated flame retardants, and provide strategies for the study of potential adverse pregnancy findings following exposure.

## Abbreviations

PCB, Polychlorinated biphenyl; ∑PCBTotal, Sum of all PCBs; ∑PCBmono, Sum of mono-ortho-substituted PCBs; ∑PCBdi,, Sum of di-ortho substituted PCBs; ∑PCBtri, Sum of tri-ortho substituted PCBs; ∑PCB1b, Sum of weak phenobarbital inducers, persistent PCBs; ∑PCB2b, Sum of limited dioxin activity, persistent PCBs; ∑PCB3, Sum of Phenobarbital, CYP1A and CYP2B inducers, biologically persistent PCBs; tC, Cholesterol; tG, Triglyceride; tN, Trans nonachlor; LOD, Limit of detection; SGA, Small-for-gestational-age; LMP, Last menstrual period.

## Competing interests

The authors declare they have no competing financial interests.

## Authors’ contributions

PFL, BC, PC designed the study and supervised the study operations, PFL, BC, PC designed and oversaw implementation of research study. MP, OIK, YW, J-SP and GB conducted laboratory analyses and contributed to the content of the manuscript. KK carried out statistical analyses under supervision of PFL and XL and wrote the manuscript under supervision of PFL. All authors provided edits and comments to the manuscript. All authors read and approved the final manuscript. Additionally, PFL: principal investigator on grant, conceived of project, oversaw all stages of analysis, significant contributions to writing and revision.

## Supplementary Material

Additional file 1 **Supplementary Tables. Microsoft Word document (.doc).** Additional documentation that would otherwise appear as “data not shown” in the manuscript. Contents include the following Tables: S1: Results of a logistic regression analysis of PCB exposure and preterm delivery; S2a: Results of a cox-regression analysis of PCB exposure (continuous variables) and gestation; S2b: Results of a cox-regression analysis of PCB exposure (tertiles) and gestation; S3: Results of linear regression analyses examining PCB exposure (using natural log-transformed exposure) and gestation; S4: Results of linear regression analyses examining PCB exposure (using natural log-transformed exposure) and birth weight; S5a-c: Spearman correlation coefficients between PCB groupings (5a), PCB congeners (5b) and PCBs and DDE (5c). (DOCX 65 kb) Click here for file
