# Supplementary material for: Prenatal polychlorinated biphenyl exposure is associated with decreased gestational length but not birth weight: archived samples from the Child Health and Development Studies pregnancy cohort
Source: Environ Health. 2012 Jul 20;11:49. doi: 10.1186/1476-069X-11-49 (PMC3411417; doi:10.1186/1476-069X-11-49)
Supplement: Additional file 1 — Supplementary Tables. Microsoft Word document (.doc). Additional documentation that would otherwise appear as “data not shown” in the manuscript. Contents include the following Tables: S1: Results of a logistic regression analysis of PCB exposure and preterm delivery; S2a: Results of a cox-regression analysis of PCB exposure (continuous variables) and gestation; S2b: Results of a cox-regression analysis of PCB exposure (tertiles) and gestation; S3: Results of linear regression analyses examining PCB exposure (using natural log-transformed exposure) and gestation; S4: Results of linear regression analyses examining PCB exposure (using natural log-transformed exposure) and birth weight; S5a-c: Spearman correlation coefficients between PCB groupings (5a), PCB congeners (5b) and PCBs and DDE (5c). (DOCX 65 kb) [file 1476-069X-11-49-S1.docx]

**Table S1. Associations between PCB exposure and preterm delivery^#^**

|  | OR_unadjusted_^1^ | 95% CI |  | OR_adjusted_^2^ | 95% CI |
| --- | --- | --- | --- | --- | --- |
| **PCB**  **groupings** |  |  |  |  |  |
| PCB_total_ | 1.16 | (0.90, 1.50) |  | 1.12 | (0.86, 1.45) |
| PCB_mono_ | 1.27 | (0.60, 2.66) |  | 1.21 | (0.54, 2.69) |
| PCB_di_ | 1.25 | (0.88, 1.78) |  | 1.17 | (0.82, 1.66) |
| PCB_tri_ | 2.22 | (0.17, 29.19) |  | 2.59 | (0.15, 44.73) |
| PCB_1b_ | 15.02 | (0.21, >999.99) |  | 8.06 | (0.10, 663.02) |
| PCB_2b_ | 1.73 | (0.79, 3.83) |  | 1.46 | (0.65, 3.27) |
| PCB_3_ | 1.39 | (0.75, 2.57) |  | 1.28 | (0.70, 2.33) |
|  |  |  |  |  |  |
| **Individual**  **PCBs** |  |  |  |  |  |
| PCB 66 | 2.92 | (0.19, 44.62) |  | 3.40 | (0.20, 57.63) |
| PCB 74 | 1.28 | (0.14, 11.66) |  | 1.07 | (0.09, 12.74) |
| PCB 99 | 4.48 | (0.41, 48.91) |  | 3.70 | (0.30, 45.33) |
| PCB 118 | 1.60 | (0.34, 7.47) |  | 1.43 | (0.27, 7.60) |
| PCB 138 | 1.81 | (0.68, 4.84) |  | 1.46 | (0.53, 4.01) |
| PCB 153 | 1.81 | (0.61, 5.40) |  | 1.44 | (0.48, 4.26) |
| PCB 170 | 39.55 | (0.90, >999.99) |  | 19.20 | (0.45, 825.14) |
| PCB 180 | 2.42 | (0.28, 21.12) |  | 2.16 | (0.27, 17.00) |
| PCB 187 | 15.02 | (0.21, >999.99) |  | 8.06 | (0.10, 663.02) |
| PCB 194 | 83.06 | (0.02, >999.99) |  | 427.32 | (0.06, >999.99) |
| PCB 203 | 0.31 | (<0.001, >999.99) |  | 1.22 | (<0.001, >999.99) |

^#^preterm delivery defined as delivery <37 weeks gestation.

^1^Crude odds ratio for non-transformed PCB measure (adjusted: laboratory and natural log-transformed serum triglycerides and cholesterol); estimated using logistic regression analysis

^2^ Odds ratio for non-transformed PCB measure (adjusted: maternal race, age, employment status and infant sex, laboratory and natural log-transformed serum triglycerides and cholesterol); estimated using logistic regression analysis

**Table S2a. Hazard ratio estimates^#^ for associations between PCB exposure (continuous) and length of gestation (days)**

|  |  | HR_unadjusted_^1^ | 95%CI | HR_adjusted_^2^ | 95% CI |
| --- | --- | --- | --- | --- | --- |
| **Organochlorine groupings** |  |  |  |  |  |
| PCB_total_ |  | 1.06^*^ | (1.01, 1.13) | 1.06^*^ | (1.00, 1.12) |
| PCB_mono_ |  | 1.15 | (0.96, 1.38) | 1.20^+^ | (1.00, 1.45) |
| PCB_di_ |  | 1.09^*^ | (1.01, 1.18) | 1.07^+^ | (1.00, 1.16) |
| PCB_tri_ |  | 1.67^+^ | (0.98, 2.83) | 1.40 | (0.78, 2.50) |
| PCB_1b_ |  | 2.71^*^ | (1.11, 6.60) | 1.80 | (0.72, 4.54) |
| PCB_2b_ |  | 1.18^+^ | (0.99, 1.42) | 1.15 | (0.96, 1.38) |
| PCB_3_ |  | 1.17^*^ | (1.03, 1.32) | 1.14^*^ | (1.03, 1.29) |
|  |  |  |  |  |  |
| **Individual Organochlorines** |  |  |  |  |  |
| PCB 66 |  | 1.21 | (0.82, 1.79) | 1.42^+^ | (0.95, 2.12) |
| PCB 74 |  | 1.42 | (0.74, 2.75) | 1.53 | (0.80, 2.94) |
| PCB 99 |  | 1.51 | (0.85, 2.65) | 1.32 | (0.75, 2.33) |
| PCB 118 |  | 1.29 | (0.92, 1.81) | 1.28 | (0.91, 1.80) |
| PCB 138 |  | 1.21 | (0.96, 1.51) | 1.18^*^ | (0.98, 1.43) |
| PCB 153 |  | 1.32^*^ | (1.04, 1.67) | 1.27^*^ | (1.01, 1.60) |
| PCB 170 |  | 2.84^*^ | (1.12, 7.24) | 2.30^+^ | (0.91, 5.84) |
| PCB 180 |  | 1.72^*^ | (1.13, 2.60) | 1.63^*^ | (1.06, 2.52) |
| PCB 187 |  | 2.71^*^ | (1.11, 6.60) | 1.80 | (0.72, 4.54) |
| PCB 194 |  | 5.74^+^ | (0.85, 38.67) | 4.67 | (0.60, 36.28) |
| PCB 203 |  | 1.79 | (0.63, 5.13) | 1.55 | (0.47, 5.17) |

^#^Hazard ratio estimate derived from cox-proportional hazard regression analysis.

^1^Hazard ratio estimate for a unit increase in the non-transformed PCB measure (adjusted: laboratory, natural log-transformed serum triglycerides and cholesterol).

^2^Hazard ratio estimate for a unit increase in the non-transformed PCB measure (adjusted: maternal race, age (continuous), employment status and infant sex, laboratory and natural log-transformed serum triglycerides and cholesterol).

^+^significant at 0.05<p<0.10

^*^significant at p<0.05

**S2b. Hazard ratio estimates^#^ for associations between PCB exposure (tertiles) and length of gestation (days)**

|  |  | Unadjusted^1^ | | | |  | Adjusted^2^ | | | |
| --- | --- | --- | --- | --- | --- | --- | --- | --- | --- | --- |
|  |  | HR_1_^3^ | 95% CI | HR_2_^4^ | 95% CI |  | HR_1_ | 95% CI | HR_2_ | 95% CI |
| **PCB tertiles** |  |  |  |  |  |  |  |  |  |  |
| PCB_total_ |  | 0.99 | (0.80, 1.23) | 1.18^*^ | (0.94, 1.49) |  | 0.97 | (0.78, 1.20) | 1.26^*^ | (1.02, 1.57) |
| PCB_mono_ |  | 1.03 | (0.84, 1.26) | 0.99 | (0.79, 1.24) |  | 1.12 | (0.90, 1.38) | 1.09 | (0.88, 1.36) |
| PCB_di_ |  | 0.94 | (0.96, 1.16) | 1.13^+^ | (0.90, 1.43) |  | 0.99 | (0.80, 1.23) | 1.29^*^ | (1.04, 1.59) |
| PCB_tri_ |  | 1.03 | (0.83, 1.28) | 1.13^+^ | (0.89, 1.43) |  | 1.05 | (0.85, 1.30) | 1.13 | (0.90, 1.42) |
| PCB_1b_ |  | 1.01 | (0.82, 1.25) | 1.15^+^ | (0.90, 1.45) |  | 1.00 | (0.80, 1.23) | 1.23^+^ | (0.98, 1.55) |
| PCB_2b_ |  | 0.92 | (0.75, 1.14) | 1.08 | (0.86, 1.35) |  | 0.99 | (0.80, 1.22) | 1.21^+^ | (0.98, 1.50) |
| PCB_3_ |  | 1.13 | (0.94, 1.40) | 1.37^**^ | (1.08, 1.72) |  | 1.12 | (0.91, 1.39) | 1.41^**^ | (1.14, 1.75) |

^#^Hazard ratio estimated derived from cox-proportional hazard regression analysis.

^1^Hazard ratio estimate examining tertiles of PCB exposure (adjusted: laboratory, natural log-transformed serum triglycerides and cholesterol).

^2^Hazard ratio estimate examining tertiles of PCB exposure (adjusted: maternal race, age (continuous), employment status and infant sex, laboratory and natural log-transformed serum triglycerides and cholesterol).

^3^Hazard ratio estimate examining the middle tertile of PCB exposure vs the lowest tertile of PCB exposure

^4^Hazard ratio estimate examining the highest tertile of PCB exposure vs the lowest tertile of PCB exposure

^+^significant at 0.05<p<0.10

^*^significant at p=0.05

^**^significant at p=0.01

**Table S3. Associations between natural log-transformed PCB exposure and length of gestation**

|  | **Unadjusted** | |  | **Adjusted** | |  | **Further adjusted for *p,p′*-DDE** | |
| --- | --- | --- | --- | --- | --- | --- | --- | --- |
|  | b_unadjusted_^1^ | 95%CI |  | b_adjusted_^2^ | 95% CI |  | b_unadjusted_^3^ | 95%CI |
| **PCB**  **groupings** |  |  |  |  |  |  |  |  |
| PCB_total_ | -0.34^+^ | (-0.69, 0.004) |  | -0.37^*^ | (-0.72, -0.02) |  | -0.33 | (-0.74, 0.071) |
| PCB_mono_ | -0.23 | (-0.54, 0.08) |  | -0.26^+^ | (-0.57, 0.05) |  | -0.22 | (-0.55, 0.12) |
| PCB_di_ | -0.34^+^ | (-0.68, 0.006) |  | -0.33^+^ | (-0.68, 0.008) |  | -0.30 | (-0.69, 0.098) |
| PCB_tri_ | -0.19 | (-0.48, 0.10) |  | -0.24 | (-0.54, 0.05) |  | -0.20 | (-0.53, 0.14) |
| PCB_1b_ | -0.27^+^ | (-0.56, 0.02) |  | -0.28^+^ | (-0.58, 0.02) |  | -0.24 | (-0.57, 0.089) |
| PCB_2b_ | -0.30^+^ | (-0.62, 0.010) |  | -0.28^+^ | (-0.60, 0.03) |  | -0.24 | (-0.60, 0.12) |
| PCB_3_ | -0.32^+^ | (-0.67, 0.03) |  | -0.35^+^ | (-0.69, 0.04) |  | -0.31 | (-0.71, 0.097) |
|  |  |  |  |  |  |  |  |  |
| **Individual**  **PCBs** |  |  |  |  |  |  |  |  |
| PCB 66 | -0.13 | (-0.32, 0.06) |  | -0.17 | (-0.37, 0.02) |  | -0.16 | (-0.35, 0.042) |
| PCB 74 | -0.15 | (-0.40, 0.10) |  | -0.13 | (-0.38, 0.12) |  | -0.075 | (-0.35, 0.20) |
| PCB 99 | -0.10 | (-0.31, 0.10) |  | -0.089 | (-0.30, 0.12) |  | -0.048 | (-0.27, 0.17) |
| PCB 118 | -0.18 | (-0.49, 0.14) |  | -0.20 | (-0.52, 0.12) |  | -0.14 | (-0.49, 0.20) |
| PCB 138 | -0.28^+^ | (-0.59, 0.04) |  | -0.25 | (-0.56, 0.07) |  | -0.20 | (-0.55, 0.16) |
| PCB 153 | -0.34^+^ | (-0.68, 0.0003) |  | -0.33^+^ | (-0.67, 0.009) |  | -0.29 | (-0.68, 0.096) |
| PCB 170 | -0.22 | (-0.49, 0.04) |  | -0.24^+^ | (-0.50, 0.03) |  | -0.20 | (-0.49, 0.097) |
| PCB 180 | -0.27 | (-0.62, 0.08) |  | -0.34^+^ | (-0.69, 0.007) |  | -0.31 | (-0.71, 0.097) |
| PCB 187 | -0.27^+^ | (-0.56, 0.02) |  | -0.28^+^ | (-0.58, 0.0) |  | -0.24 | (-0.57, 0.089) |
| PCB 194 | -0.05 | (-0.36, 0.26) |  | -0.19 | (-0.51, 0.13) |  | -0.17 | (-0.52, 0.18) |
| PCB 203 | 0.08 | (-0.18, 0.34) |  | -0.029 | (-0.30, 0.24) |  | 0.047 | (-0.25, 0.34) |

^1^Parameter estimate for natural log-transformed PCB measure (adjusted: laboratory, natural log-transformed serum triglycerides and cholesterol).

^2^Parameter estimate for natural log-transformed PCB measure (adjusted: maternal race, age, employment status and infant sex, laboratory and natural log-transformed serum triglycerides and cholesterol)

^3^Parameter estimate for natural log-transformed PCB measure (adjusted: *p,p′-*DDE, maternal race, age, employment status and infant sex, laboratory and natural log-transformed serum triglycerides and cholesterol)

^+^significant at 0.05<p<0.10; ^*^significant at p<0.05

**Table S4.** **Associations between natural log-transformed PCB exposure and birth weight**

|  | **Unadjusted** | |  | **Adjusted** | |  | **Further adjusted for *p,p′*-DDE** | |
| --- | --- | --- | --- | --- | --- | --- | --- | --- |
|  | b_unadjusted_^1^ | 95%CI |  | b_adjusted_^2^ | 95% CI |  | b_unadjusted_^3^ | 95%CI |
| **PCB**  **groupings** |  |  |  |  |  |  |  |  |
| PCB_total_ | -6 | (-114, 102) |  | 57 | (-42, 157) |  | 80 | (-34, 194) |
| PCB_mono_ | 46 | (-48, 140) |  | 63 | (-23, 149) |  | 77 | (-17, 171) |
| PCB_di_ | -33 | (-139, 73) |  | 43 | (-54, 141) |  | 61 | (-51, 173) |
| PCB_tri_ | 9 | (-81, 99) |  | 53 | (-33, 138) |  | 69 | (-26, 164) |
| PCB_1b_ | -23 | (-112, 66) |  | 32 | (-52, 116) |  | 43 | (-50, 136) |
| PCB_2b_ | -20 | (-117, 78) |  | 50 | (-40, 139) |  | 67 | (-34, 169) |
| PCB_3_ | -29 | (-137, 80) |  | 43 | (-57, 143) |  | 62 | (-54, 177) |
|  |  |  |  |  |  |  |  |  |
| **Individual**  **PCBs** |  |  |  |  |  |  |  |  |
| PCB 66 | 35 | (-24, 94) |  | 19 | (-35, 73) |  | 21 | (-35, 77) |
| PCB 74 | -21 | (-97, 56) |  | 19 | (-49, 88) |  | 26 | (-49, 100) |
| PCB 99 | -46 | (-111, 20) |  | -8 | (-68, 51) |  | -8 | (-72, 55) |
| PCB 118 | 45 | (-53, 143) |  | 56 | (-33, 146) |  | 66 | (-30, 162) |
| PCB 138 | -2 | (-98, 95) |  | 60 | (-29, 149) |  | 79 | (-21, 180) |
| PCB 153 | -59 | (-164, 46) |  | 13 | (-83, 110) |  | 20 | (-90, 130) |
| PCB 170 | -42 | (-125, 41) |  | 12 | (-65, 88) |  | 16 | (-67, 100) |
| PCB 180 | 11 | (-98, 119) |  | 74 | (-27, 175) |  | 101^+^ | (-15, 216) |
| PCB 187 | -23 | (-112, 66) |  | 32 | (-52, 116) |  | 43 | (-50, 136) |
| PCB 194 | 34 | (-61, 129) |  | 65 | (-28, 159) |  | 99^+^ | (-2, 201) |
| PCB 203 | 64 | (-17, 145) |  | 66 | (-12, 144)^+^ |  | 80^+^ | (-5, 165) |

^1^Parameter estimate for natural log-transformed PCB measure (adjusted: laboratory, natural log-transformed serum triglycerides and cholesterol).

^2^Parameter estimate for natural log-transformed PCB measure (adjusted: maternal race, age, smoking status and BMI, infant sex, length of gestation (centered), quadratic length of gestation (centered), laboratory and natural log-transformed serum triglycerides and cholesterol).

^3^Parameter estimate for natural log-transformed PCB measure (adjusted: *p,p′-*DDE, maternal race, age, smoking status and BMI, infant sex, length of gestation (centered), quadratic length of gestation (centered), laboratory and natural log-transformed serum triglycerides and cholesterol).

^+^significant at 0.05<p<0.10

**Table S5a. Spearman correlations between PCB groupings**

|  | **PCB_total_** | **PCB_mono_** | **PCB_di_** | **PCB_tri_** | **PCB_1b_** | **PCB_2b_** | **PCB_3_** |
| --- | --- | --- | --- | --- | --- | --- | --- |
| **PCB_total_** | ***1.000*** | 0.840 | 0.968 | 0.832 | 0.821 | 0.934 | 0.966 |
|  |  | <.0001 | <.0001 | <.0001 | <.0001 | <.0001 | <.0001 |
|  | 578 | 578 | 578 | 578 | 578 | 578 | 578 |
| **PCB_mono_** | 0.840 | ***1.000*** | 0.716 | 0.597 | 0.565 | 0.692 | 0.712 |
|  | <.0001 |  | <.0001 | <.0001 | <.0001 | <.0001 | <.0001 |
|  | 578 | 578 | 578 | 578 | 578 | 578 | 578 |
| **PCB_di_** | 0.968 | 0.716 | ***1.000*** | 0.818 | 0.826 | 0.972 | 0.981 |
|  | <.0001 | <.0001 |  | <.0001 | <.0001 | <.0001 | <.0001 |
|  | 578 | 578 | 578 | 578 | 578 | 578 | 578 |
| **PCB_tri_** | 0.832 | 0.597 | 0.818 | ***1.000*** | 0.947 | 0.754 | 0.869 |
|  | <.0001 | <.0001 | <.0001 |  | <.0001 | <.0001 | <.0001 |
|  | 578 | 578 | 578 | 578 | 578 | 578 | 578 |
| **PCB_1b_** | 0.821 | 0.565 | 0.826 | 0.947 | ***1.000*** | 0.779 | 0.851 |
|  | <.0001 | <.0001 | <.0001 | <.0001 |  | <.0001 | <.0001 |
|  | 578 | 578 | 578 | 578 | 578 | 578 | 578 |
| **PCB_2b_** | 0.934 | 0.692 | 0.972 | 0.754 | 0.779 | ***1.000*** | 0.918 |
|  | <.0001 | <.0001 | <.0001 | <.0001 | <.0001 |  | <.0001 |
|  | 578 | 578 | 578 | 578 | 578 | 578 | 578 |
| **PCB_3_** | 0.966 | 0.712 | 0.981 | 0.869 | 0.851 | 0.918 | ***1.000*** |
|  | <.0001 | <.0001 | <.0001 | <.0001 | <.0001 | <.0001 |  |
|  | 578 | 578 | 578 | 578 | 578 | 578 | 578 |

**Table S5b. Spearman correlations between PCB congeners**

|  | **66** | **74** | **99** | **118** | **138** | **153** | **170** | **180** | **187** | **194** | **203** |
| --- | --- | --- | --- | --- | --- | --- | --- | --- | --- | --- | --- |
| **66** | ***1.000*** | 0.490 | 0.345 | 0.592 | 0.347 | 0.461 | 0.352 | 0.257 | 0.382 | 0.305 | 0.393 |
|  |  | <.0001 | <.0001 | <.0001 | <.0001 | <.0001 | <.0001 | <.0001 | <.0001 | <.0001 | <.0001 |
|  | 578 | 578 | 578 | 578 | 578 | 578 | 578 | 578 | 578 | 459 | 578 |
| **74** | 0.490 | ***1.000*** | 0.652 | 0.651 | 0.593 | 0.563 | 0.490 | 0.501 | 0.445 | 0.456 | 0.430 |
|  | <.0001 |  | <.0001 | <.0001 | <.0001 | <.0001 | <.0001 | <.0001 | <.0001 | <.0001 | <.0001 |
|  | 578 | 578 | 578 | 578 | 578 | 578 | 578 | 578 | 578 | 459 | 578 |
| **99** | 0.345 | 0.652 | ***1.000*** | 0.743 | 0.740 | 0.676 | 0.590 | 0.577 | 0.543 | 0.518 | 0.466 |
|  | <.0001 | <.0001 |  | <.0001 | <.0001 | <.0001 | <.0001 | <.0001 | <.0001 | <.0001 | <.0001 |
|  | 578 | 578 | 578 | 578 | 578 | 578 | 578 | 578 | 578 | 459 | 578 |
| **118** | 0.592 | 0.651 | 0.743 | ***1.000*** | 0.792 | 0.775 | 0.665 | 0.627 | 0.622 | 0.516 | 0.552 |
|  | <.0001 | <.0001 | <.0001 |  | <.0001 | <.0001 | <.0001 | <.0001 | <.0001 | <.0001 | <.0001 |
|  | 578 | 578 | 578 | 578 | 578 | 578 | 578 | 578 | 578 | 459 | 578 |
| **138** | 0.347 | 0.593 | 0.740 | 0.792 | ***1.000*** | 0.905 | 0.831 | 0.794 | 0.746 | 0.632 | 0.540 |
|  | <.0001 | <.0001 | <.0001 | <.0001 |  | <.0001 | <.0001 | <.0001 | <.0001 | <.0001 | <.0001 |
|  | 578 | 578 | 578 | 578 | 578 | 578 | 578 | 578 | 578 | 459 | 578 |
| **153** | 0.461 | 0.563 | 0.676 | 0.775 | 0.905 | ***1.000*** | 0.882 | 0.832 | 0.822 | 0.695 | 0.657 |
|  | <.0001 | <.0001 | <.0001 | <.0001 | <.0001 |  | <.0001 | <.0001 | <.0001 | <.0001 | <.0001 |
|  | 578 | 578 | 578 | 578 | 578 | 578 | 578 | 578 | 578 | 459 | 578 |
| **170** | 0.352 | 0.490 | 0.590 | 0.665 | 0.831 | 0.882 | ***1.000*** | 0.860 | 0.811 | 0.791 | 0.700 |
|  | <.0001 | <.0001 | <.0001 | <.0001 | <.0001 | <.0001 |  | <.0001 | <.0001 | <.0001 | <.0001 |
|  | 578 | 578 | 578 | 578 | 578 | 578 | 578 | 578 | 578 | 459 | 578 |
| **180** | 0.257 | 0.501 | 0.577 | 0.627 | 0.794 | 0.832 | 0.860 | ***1.000*** | 0.824 | 0.897 | 0.715 |
|  | <.0001 | <.0001 | <.0001 | <.0001 | <.0001 | <.0001 | <.0001 |  | <.0001 | <.0001 | <.0001 |
|  | 578 | 578 | 578 | 578 | 578 | 578 | 578 | 578 | 578 | 459 | 578 |
| **187** | 0.382 | 0.445 | 0.543 | 0.622 | 0.746 | 0.822 | 0.811 | 0.824 | ***1.000*** | 0.784 | 0.722 |
|  | <.0001 | <.0001 | <.0001 | <.0001 | <.0001 | <.0001 | <.0001 | <.0001 |  | <.0001 | <.0001 |
|  | 578 | 578 | 578 | 578 | 578 | 578 | 578 | 578 | 578 | 459 | 578 |
| **194** | 0.305 | 0.456 | 0.518 | 0.516 | 0.632 | 0.695 | 0.791 | 0.897 | 0.784 | ***1.000*** | 0.886 |
|  | <.0001 | <.0001 | <.0001 | <.0001 | <.0001 | <.0001 | <.0001 | <.0001 | <.0001 |  | <.0001 |
|  | 459 | 459 | 459 | 459 | 459 | 459 | 459 | 459 | 459 | 459 | 459 |
| **203** | 0.393 | 0.430 | 0.466 | 0.552 | 0.540 | 0.657 | 0.700 | 0.715 | 0.722 | 0.886 | ***1.000*** |
|  | <.0001 | <.0001 | <.0001 | <.0001 | <.0001 | <.0001 | <.0001 | <.0001 | <.0001 | <.0001 |  |
|  | 578 | 578 | 578 | 578 | 578 | 578 | 578 | 578 | 578 | 459 | 578 |

**Table S5c. Spearman correlations between PCBs (individual congeners and groupings) and p,p′-DDE.**

| **PCB groupings:** | |  | **Individual Congeners** | |
| --- | --- | --- | --- | --- |
|  | **p,p′-DDE** |  |  | **p,p′-DDE** |
| **PCB_total_** | 0.588 |  | **66** | 0.313 |
|  | <.0001 |  |  | <.0001 |
|  | 577 |  |  | 577 |
| **PCB_mono_** | 0.514 |  | **74** | 0.528 |
|  | <.0001 |  |  | <.0001 |
|  | 577 |  |  | 577 |
| **PCB_di_** | 0.577 |  | **99** | 0.528 |
|  | <.0001 |  |  | <.0001 |
|  | 577 |  |  | 577 |
| **PCB_tri_** | 0.547 |  | **118** | 0.505 |
|  | <.0001 |  |  | <.0001 |
|  | 577 |  |  | 577 |
| **PCB_1b_** | 0.510 |  | **138** | 0.543 |
|  | <.0001 |  |  | <.0001 |
|  | 577 |  |  | 578 |
| **PCB_2b_** | 0.543 |  | **153** | 0.578 |
|  | <.0001 |  |  | <.0001 |
|  | 577 |  |  | 578 |
| **PCB_3_** | 0.587 |  | **170** | 0.499 |
|  | <.0001 |  |  | <.0001 |
|  | 577 |  |  | 577 |
|  |  |  | **180** | 0.523 |
|  |  |  |  | <.0001 |
|  |  |  |  | 578 |
|  |  |  | **187** | 0.51 |
|  |  |  |  | <.0001 |
|  |  |  |  | 577 |
|  |  |  | **194** | 0.462 |
|  |  |  |  | <.0001 |
|  |  |  |  | 458 |
|  |  |  | **203** | 0.523 |
|  |  |  |  | <.0001 |
|  |  |  |  | 577 |
